# Supplementary material for: Shared Treg dysregulation underlies psoriasis vulgaris and atopic dermatitis through the PHF19-PRC2 epigenetic axis
Source: Front Immunol. 2026 Aug 20;17:1932788. doi: 10.3389/fimmu.2026.1932788 (PMC13538097; doi:10.3389/fimmu.2026.1932788)
Supplement: Supplementary file 3 [file DataSheet3.zip › Additional files/Additional file 2/20.MTR.forest.pdf]

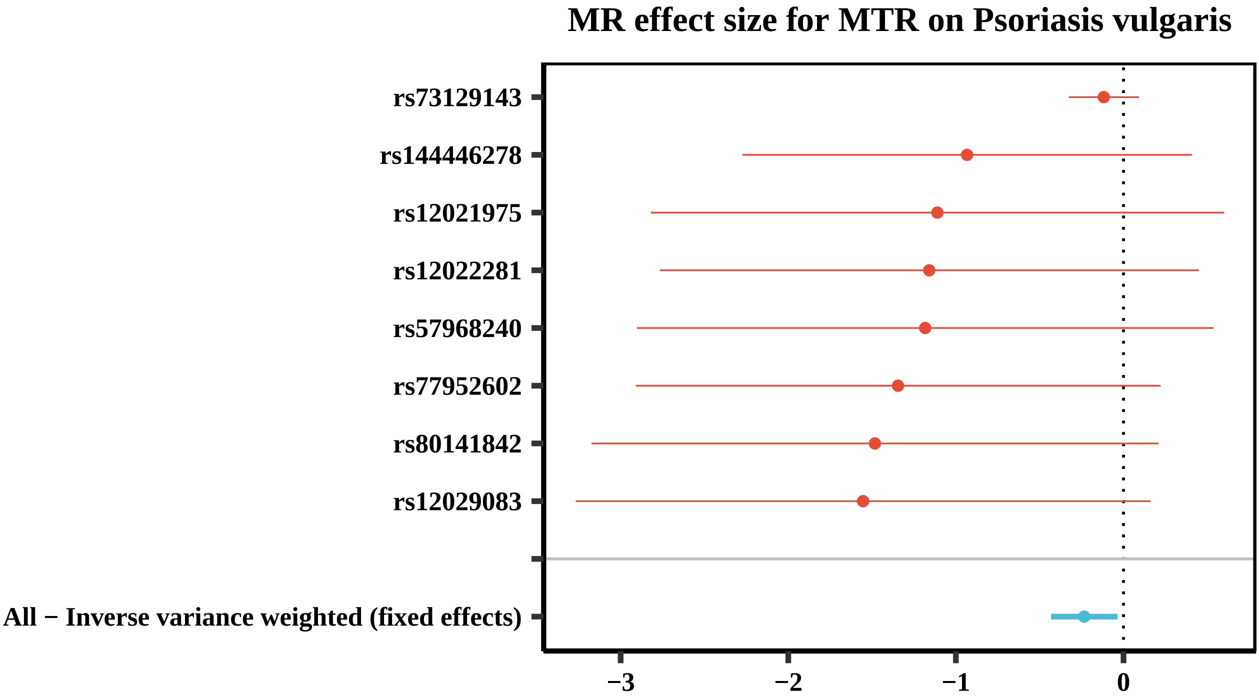
MR effect size for MTR on Psoriasis vulgaris
rs73129143
rs144446278
rs12021975
rs12022281
rs57968240
rs77952602
rs80141842
rs12029083
All − Inverse variance weighted (fixed effects)
−3
−2
−1
0
